# 1,3-Bis(4-*tert*-butyl­benz­yl)pyrimidine-2,4(1*H*,3*H*)-dione

**DOI:** 10.1107/S160053681102054X

**Published:** 2011-06-11

**Authors:** Gong-Chun Li, Hong-Sheng Wang, Feng-Xiang Zhu, Yu-Jiao Niu

**Affiliations:** a College of Chemistry and Chemical Engineering, Xuchang University, Xuchang, Henan Province 461000, People’s Republic of China

## Abstract

In the crystal structure of the title mol­ecule, C_26_H_32_N_2_O_2_, the six methyl groups are disordered over two positions, with site-occupancy ratios of 0.665 (8):0.335 (8) and 0.639 (8):0.361 (8). The central pyrimidine ring is almost planar with an r.m.s. deviation of 0.009 Å. The dihedral angles formed by the two benzene rings with the pyrimidine ring are 70.70 (8) and 88.02 (9)°. The dihedral angle between two benzene rings is 46.67 (10)°.

## Related literature

For the applications of pyrimidine derivatives as pesticides and pharmaceutical agents, see: Condon *et al.* (1993 ▶); as agrochemicals, see: Maeno *et al.* (1990 ▶); as anti­viral agents, see: Gilchrist (1997 ▶); as herbicides, see: Selby *et al.* (2002 ▶). For a related structure, see: Yang & Li (2006 ▶).
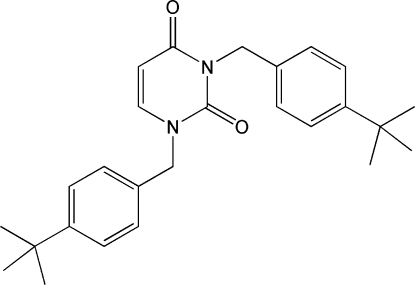


## Experimental

### 

#### Crystal data

C_26_H_32_N_2_O_2_
*M* *_r_* = 404.54
Triclinic, 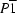

*a* = 7.5742 (18) Å
*b* = 12.191 (3) Å
*c* = 13.080 (3) Å
α = 88.643 (4)°
β = 84.809 (4)°
γ = 78.381 (4)°
*V* = 1178.2 (5) Å^3^
*Z* = 2
Mo *K*α radiation
μ = 0.07 mm^−1^
*T* = 294 K
0.26 × 0.24 × 0.14 mm

#### Data collection

Bruker SMART 1000 diffractometer
Absorption correction: multi-scan (*SADABS*; Sheldrick, 1996 ▶) *T*_min_ = 0.982, *T*_max_ = 0.990
5993 measured reflections
4127 independent reflections
2609 reflections with *I* > 2σ(*I*)
*R*_int_ = 0.019

#### Refinement

*R*[*F* ^2^ > 2σ(*F* ^2^)] = 0.056
*wR*(*F* ^2^) = 0.198
*S* = 1.09
4127 reflections
328 parameters
139 restraints
H-atom parameters constrained
Δρ_max_ = 0.38 e Å^−3^
Δρ_min_ = −0.28 e Å^−3^

### 

Data collection: *SMART* (Bruker, 1999 ▶); cell refinement: *SAINT* (Bruker, 1999 ▶); data reduction: *SAINT*; program(s) used to solve structure: *SHELXS97* (Sheldrick, 2008 ▶); program(s) used to refine structure: *SHELXL97* (Sheldrick, 2008 ▶); molecular graphics: *SHELXTL* (Sheldrick, 2008 ▶); software used to prepare material for publication: *SHELXL97*.

## Supplementary Material

Crystal structure: contains datablock(s) global, I. DOI: 10.1107/S160053681102054X/om2436sup1.cif

Structure factors: contains datablock(s) I. DOI: 10.1107/S160053681102054X/om2436Isup2.hkl

Supplementary material file. DOI: 10.1107/S160053681102054X/om2436Isup3.cml

Additional supplementary materials:  crystallographic information; 3D view; checkCIF report

## Figures and Tables

**Fig. 1.**
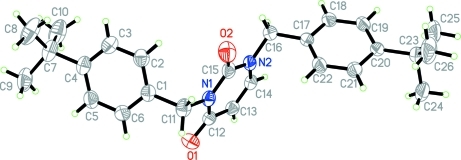
The asymmetric unit of the title compound with displacement ellipsoids drawn at the 30% probability level.

## supplementary crystallographic information

### Comment

Pyrimidine derivatives are very important molecules in biology and have many application in the areas of pesticide and pharmaceutical agents (Condon *et al.*, 1993). For example, imazosulfuron, ethirmol and mepanipyrim have been commercialized as agrochemicals (Maeno *et al.*, 1990). Pyrimidine derivatives have also been developed as antiviral agents, such as AZT, which is the most widely used anti-AIDS drug (Gilchrist, 1997). Recently, a new series of highly active herbicides of substituted azolylpyrimidines were reported (Selby *et al.*, 2002). In order to discover further biologically active pyrimidine compounds, the title compound was synthesized and its crystal structure determined (Fig. 1).

In the crystal structure of the title molecule, the six methyl groups show positional disorder, the occupancy factors of two possible sites, C8/C9/C10 and C8'/C9'/C10', were refined to 0.665 (8) and 0.335 (8), respectively, C24/C25/C26 and C24'/C25'/C26', were refined to 0.639 (8) and 0.361 (8), respectively. For a crystal structure related to the title compound, see: Yang & Li, 2006.

### Experimental

Uracil (0.56 g, 5 mmol) and anhydrous potassium carbonate (0.84 g, 6 mmol) were mixed in *N*,*N*-dimethylformamide (20 ml). A solution of 4-tertbutylbenzyl chloride (0.92 g, 5 mmol) in acetone (10 ml) was then added dropwise, with stirring, at room temperature, and the mixture was stirred for another 10 h and then refluxed for 4 h. The solvent was evaporated *in vacuo* and the residue was washed with water. The resulting white precipitate was filtered off and purified by column chromatography on silica gel (petroleum ether:ethyl acetate = 2:1). The title compound was recrystallized from ethanol and single crystals were obtained.

### Refinement

All H atoms were placed in calculated positions, with C—H(aromatic) = 0.93 Å and C—H(aliphatic) = 0.96 Å or 0.97 Å, and included in the final cycles of refinement using a riding model, with *U*_iso_(H) = 1.2U_eq_(C).

### Crystal data

**Table:**

C_26_H_32_N_2_O_2_	*Z* = 2
*M**_r_* = 404.54	*F*(000) = 436
Triclinic, *P*1	*D*_x_ = 1.140 Mg m^−^^3^
*a* = 7.5742 (18) Å	Mo *K*α radiation, λ = 0.71073 Å
*b* = 12.191 (3) Å	Cell parameters from 2134 reflections
*c* = 13.080 (3) Å	θ = 2.3–25.7°
α = 88.643 (4)°	µ = 0.07 mm^−^^1^
β = 84.809 (4)°	*T* = 294 K
γ = 78.381 (4)°	Prism, colourless
*V* = 1178.2 (5) Å^3^	0.26 × 0.24 × 0.14 mm

### Data collection

**Table:**

Bruker SMART 1000 diffractometer	4127 independent reflections
Radiation source: fine-focus sealed tube	2609 reflections with *I* > 2σ(*I*)
graphite	*R*_int_ = 0.019
φ and ω scans	θ_max_ = 25.0°, θ_min_ = 1.7°
Absorption correction: multi-scan (*SADABS*; Sheldrick, 1996)	*h* = −8→8
*T*_min_ = 0.982, *T*_max_ = 0.990	*k* = −14→8
5993 measured reflections	*l* = −15→14

### Refinement

**Table:**

Refinement on *F*^2^	Primary atom site location: structure-invariant direct methods
Least-squares matrix: full	Secondary atom site location: difference Fourier map
*R*[*F*^2^ > 2σ(*F*^2^)] = 0.056	Hydrogen site location: inferred from neighbouring sites
*wR*(*F*^2^) = 0.198	H-atom parameters constrained
*S* = 1.09	*w* = 1/[σ^2^(*F*_o_^2^) + (0.0995*P*)^2^ + 0.2026*P*] where *P* = (*F*_o_^2^ + 2*F*_c_^2^)/3
4127 reflections	(Δ/σ)_max_ = 0.011
328 parameters	Δρ_max_ = 0.38 e Å^−^^3^
139 restraints	Δρ_min_ = −0.28 e Å^−^^3^

### Special details

**Table:**

Geometry. All e.s.d.'s (except the e.s.d. in the dihedral angle between two l.s. planes) are estimated using the full covariance matrix. The cell e.s.d.'s are taken into account individually in the estimation of e.s.d.'s in distances, angles and torsion angles; correlations between e.s.d.'s in cell parameters are only used when they are defined by crystal symmetry. An approximate (isotropic) treatment of cell e.s.d.'s is used for estimating e.s.d.'s involving l.s. planes.
Refinement. Refinement of *F*^2^ against ALL reflections. The weighted *R*-factor *wR* and goodness of fit *S* are based on *F*^2^, conventional *R*-factors *R* are based on *F*, with *F* set to zero for negative *F*^2^. The threshold expression of *F*^2^ > σ(*F*^2^) is used only for calculating *R*-factors(gt) *etc*. and is not relevant to the choice of reflections for refinement. *R*-factors based on *F*^2^ are statistically about twice as large as those based on *F*, and *R*-factors based on ALL data will be even larger.

### Fractional atomic coordinates and isotropic or equivalent isotropic displacement parameters (Å^2^)

**Table:**

	*x*	*y*	*z*	*U*_iso_*/*U*_eq_	Occ. (<1)
O1	0.3639 (3)	0.35436 (17)	0.03048 (17)	0.0891 (7)	
O2	0.1481 (3)	0.70375 (17)	0.16032 (16)	0.0918 (7)	
N1	0.2605 (3)	0.52942 (18)	0.09502 (16)	0.0584 (6)	
N2	0.1110 (3)	0.67313 (17)	−0.00641 (16)	0.0602 (6)	
C1	0.1889 (4)	0.4353 (2)	0.2598 (2)	0.0615 (7)	
C2	0.0327 (4)	0.4990 (2)	0.3074 (2)	0.0722 (8)	
H2	0.0103	0.5765	0.2999	0.087*	
C3	−0.0894 (4)	0.4494 (3)	0.3655 (2)	0.0747 (8)	
H3	−0.1928	0.4945	0.3971	0.090*	
C4	−0.0645 (4)	0.3340 (3)	0.37905 (19)	0.0673 (8)	
C5	0.0912 (4)	0.2725 (3)	0.3311 (2)	0.0766 (8)	
H5	0.1136	0.1949	0.3381	0.092*	
C6	0.2160 (4)	0.3216 (2)	0.2728 (2)	0.0726 (8)	
H6	0.3202	0.2767	0.2419	0.087*	
C7	−0.2038 (4)	0.2787 (3)	0.4413 (2)	0.0854 (10)	
C8	−0.2405 (13)	0.3221 (8)	0.5462 (4)	0.124 (3)	0.665 (8)
H8A	−0.1549	0.2795	0.5891	0.186*	0.665 (8)
H8B	−0.3608	0.3161	0.5722	0.186*	0.665 (8)
H8C	−0.2298	0.3993	0.5460	0.186*	0.665 (8)
C9	−0.1324 (9)	0.1478 (4)	0.4520 (5)	0.113 (3)	0.665 (8)
H9A	−0.1142	0.1143	0.3852	0.170*	0.665 (8)
H9B	−0.2199	0.1159	0.4941	0.170*	0.665 (8)
H9C	−0.0200	0.1341	0.4831	0.170*	0.665 (8)
C10	−0.3628 (11)	0.2886 (9)	0.3810 (7)	0.122 (4)	0.665 (8)
H10A	−0.3357	0.2351	0.3263	0.183*	0.665 (8)
H10B	−0.3913	0.3628	0.3527	0.183*	0.665 (8)
H10C	−0.4645	0.2745	0.4248	0.183*	0.665 (8)
C8'	−0.122 (2)	0.2264 (16)	0.5340 (10)	0.140 (6)	0.335 (8)
H8'A	−0.0271	0.1639	0.5141	0.211*	0.335 (8)
H8'B	−0.2131	0.2011	0.5790	0.211*	0.335 (8)
H8'C	−0.0733	0.2805	0.5688	0.211*	0.335 (8)
C9'	−0.315 (3)	0.2241 (18)	0.3804 (18)	0.148 (9)	0.335 (8)
H9'A	−0.2398	0.1626	0.3431	0.222*	0.335 (8)
H9'B	−0.3749	0.2772	0.3329	0.222*	0.335 (8)
H9'C	−0.4045	0.1968	0.4254	0.222*	0.335 (8)
C10'	−0.3706 (19)	0.3649 (11)	0.4993 (15)	0.158 (7)	0.335 (8)
H10D	−0.3792	0.4378	0.4683	0.236*	0.335 (8)
H10E	−0.3507	0.3692	0.5704	0.236*	0.335 (8)
H10F	−0.4810	0.3391	0.4936	0.236*	0.335 (8)
C11	0.3239 (4)	0.4898 (3)	0.1953 (2)	0.0726 (8)	
H11A	0.3466	0.5527	0.2326	0.087*	
H11B	0.4373	0.4362	0.1842	0.087*	
C12	0.2895 (3)	0.4514 (2)	0.0154 (2)	0.0614 (7)	
C13	0.2250 (3)	0.4949 (2)	−0.0798 (2)	0.0616 (7)	
H13	0.2422	0.4486	−0.1369	0.074*	
C14	0.1403 (3)	0.6012 (2)	−0.0872 (2)	0.0600 (7)	
H14	0.0996	0.6275	−0.1499	0.072*	
C15	0.1721 (4)	0.6400 (2)	0.0876 (2)	0.0636 (7)	
C16	0.0077 (4)	0.7871 (2)	−0.0196 (2)	0.0716 (8)	
H16A	−0.0309	0.8199	0.0476	0.086*	
H16B	−0.1002	0.7833	−0.0530	0.086*	
C17	0.1121 (3)	0.8626 (2)	−0.08177 (19)	0.0564 (6)	
C18	0.0240 (4)	0.9452 (2)	−0.1419 (2)	0.0667 (7)	
H18	−0.0999	0.9523	−0.1455	0.080*	
C19	0.1135 (4)	1.0185 (2)	−0.1976 (2)	0.0660 (7)	
H19	0.0485	1.0735	−0.2378	0.079*	
C20	0.2971 (3)	1.0119 (2)	−0.19493 (19)	0.0561 (6)	
C21	0.3847 (4)	0.9284 (2)	−0.1336 (2)	0.0699 (8)	
H21	0.5084	0.9213	−0.1294	0.084*	
C22	0.2951 (4)	0.8550 (2)	−0.0784 (2)	0.0705 (8)	
H22	0.3595	0.7997	−0.0382	0.085*	
C23	0.3990 (4)	1.0923 (2)	−0.2568 (2)	0.0697 (8)	
C24	0.5569 (9)	1.0191 (5)	−0.3220 (6)	0.109 (3)	0.639 (8)
H24A	0.6495	0.9864	−0.2787	0.164*	0.639 (8)
H24B	0.6057	1.0646	−0.3738	0.164*	0.639 (8)
H24C	0.5138	0.9609	−0.3542	0.164*	0.639 (8)
C25	0.2801 (11)	1.1667 (8)	−0.3234 (8)	0.146 (4)	0.639 (8)
H25A	0.1830	1.2122	−0.2818	0.219*	0.639 (8)
H25B	0.2313	1.1225	−0.3691	0.219*	0.639 (8)
H25C	0.3485	1.2140	−0.3626	0.219*	0.639 (8)
C26	0.4789 (13)	1.1547 (7)	−0.1802 (6)	0.132 (3)	0.639 (8)
H26A	0.3832	1.2012	−0.1386	0.198*	0.639 (8)
H26B	0.5537	1.2006	−0.2162	0.198*	0.639 (8)
H26C	0.5507	1.1020	−0.1372	0.198*	0.639 (8)
C24'	0.381 (2)	1.0855 (14)	−0.3731 (8)	0.121 (5)	0.361 (8)
H24D	0.2676	1.1305	−0.3892	0.182*	0.361 (8)
H24E	0.3860	1.0091	−0.3914	0.182*	0.361 (8)
H24F	0.4783	1.1128	−0.4108	0.182*	0.361 (8)
C25'	0.3211 (19)	1.2150 (8)	−0.2250 (12)	0.108 (5)	0.361 (8)
H25D	0.2133	1.2181	−0.1796	0.162*	0.361 (8)
H25E	0.2927	1.2595	−0.2850	0.162*	0.361 (8)
H25F	0.4089	1.2435	−0.1905	0.162*	0.361 (8)
C26'	0.6022 (14)	1.0769 (10)	−0.2462 (11)	0.105 (4)	0.361 (8)
H26D	0.6216	1.1219	−0.1906	0.158*	0.361 (8)
H26E	0.6614	1.0997	−0.3088	0.158*	0.361 (8)
H26F	0.6511	0.9996	−0.2326	0.158*	0.361 (8)

### Atomic displacement parameters (Å^2^)

**Table:**

	*U*^11^	*U*^22^	*U*^33^	*U*^12^	*U*^13^	*U*^23^
O1	0.0871 (15)	0.0655 (14)	0.1065 (17)	0.0033 (11)	−0.0076 (12)	0.0044 (12)
O2	0.136 (2)	0.0707 (13)	0.0737 (13)	−0.0346 (13)	−0.0009 (13)	−0.0132 (11)
N1	0.0566 (13)	0.0605 (13)	0.0629 (13)	−0.0244 (10)	−0.0033 (10)	0.0047 (11)
N2	0.0676 (14)	0.0502 (12)	0.0635 (13)	−0.0174 (10)	0.0028 (11)	0.0035 (10)
C1	0.0574 (16)	0.0724 (18)	0.0609 (15)	−0.0246 (13)	−0.0145 (12)	0.0081 (13)
C2	0.082 (2)	0.0661 (18)	0.0721 (18)	−0.0243 (16)	−0.0076 (15)	−0.0004 (14)
C3	0.0740 (19)	0.088 (2)	0.0648 (17)	−0.0241 (16)	0.0013 (14)	−0.0058 (15)
C4	0.0737 (19)	0.087 (2)	0.0502 (14)	−0.0365 (16)	−0.0119 (13)	0.0047 (14)
C5	0.081 (2)	0.0698 (19)	0.083 (2)	−0.0248 (16)	−0.0104 (17)	0.0152 (16)
C6	0.0613 (17)	0.0723 (19)	0.0831 (19)	−0.0125 (14)	−0.0058 (14)	0.0108 (15)
C7	0.092 (2)	0.120 (3)	0.0588 (17)	−0.057 (2)	−0.0062 (16)	0.0071 (17)
C8	0.150 (7)	0.179 (8)	0.067 (3)	−0.100 (6)	0.024 (4)	−0.030 (4)
C9	0.151 (6)	0.099 (4)	0.100 (4)	−0.064 (4)	0.015 (4)	0.016 (3)
C10	0.111 (5)	0.185 (9)	0.101 (5)	−0.101 (6)	−0.015 (4)	0.016 (6)
C8'	0.157 (12)	0.168 (14)	0.099 (10)	−0.049 (10)	0.007 (8)	0.027 (10)
C9'	0.162 (14)	0.172 (16)	0.141 (13)	−0.106 (13)	−0.003 (10)	−0.051 (13)
C10'	0.127 (11)	0.196 (14)	0.151 (13)	−0.061 (11)	0.053 (10)	−0.033 (11)
C11	0.0662 (18)	0.090 (2)	0.0713 (18)	−0.0344 (15)	−0.0163 (14)	0.0128 (15)
C12	0.0491 (15)	0.0599 (17)	0.0756 (18)	−0.0163 (13)	0.0026 (13)	0.0055 (14)
C13	0.0624 (16)	0.0601 (17)	0.0644 (16)	−0.0200 (13)	0.0018 (13)	−0.0039 (13)
C14	0.0593 (16)	0.0651 (17)	0.0589 (15)	−0.0229 (13)	−0.0002 (12)	0.0042 (13)
C15	0.0693 (17)	0.0577 (16)	0.0688 (18)	−0.0283 (14)	0.0026 (14)	0.0006 (14)
C16	0.0684 (18)	0.0571 (17)	0.0852 (19)	−0.0105 (14)	0.0095 (15)	0.0043 (14)
C17	0.0581 (16)	0.0479 (14)	0.0614 (15)	−0.0094 (12)	0.0023 (12)	−0.0033 (12)
C18	0.0536 (15)	0.0588 (16)	0.0867 (19)	−0.0098 (13)	−0.0069 (14)	0.0056 (14)
C19	0.0629 (17)	0.0542 (15)	0.0786 (18)	−0.0055 (13)	−0.0113 (14)	0.0114 (13)
C20	0.0596 (16)	0.0470 (14)	0.0602 (15)	−0.0095 (12)	0.0000 (12)	−0.0027 (11)
C21	0.0542 (16)	0.0687 (18)	0.088 (2)	−0.0142 (14)	−0.0098 (14)	0.0112 (15)
C22	0.0657 (18)	0.0637 (17)	0.0810 (19)	−0.0099 (14)	−0.0134 (14)	0.0218 (14)
C23	0.0698 (19)	0.0582 (16)	0.0801 (19)	−0.0174 (14)	0.0087 (15)	0.0017 (14)
C24	0.118 (5)	0.090 (4)	0.114 (5)	−0.035 (4)	0.045 (4)	0.009 (4)
C25	0.118 (6)	0.134 (7)	0.181 (9)	−0.031 (5)	−0.006 (6)	0.099 (7)
C26	0.170 (8)	0.106 (6)	0.142 (6)	−0.094 (6)	0.018 (5)	−0.013 (5)
C24'	0.158 (12)	0.160 (12)	0.068 (6)	−0.094 (10)	−0.001 (7)	0.018 (7)
C25'	0.123 (9)	0.053 (5)	0.141 (10)	−0.024 (6)	0.036 (8)	0.011 (6)
C26'	0.083 (7)	0.097 (8)	0.137 (10)	−0.028 (6)	0.002 (6)	0.013 (7)

### Geometric parameters (Å, °)

**Table:**

O1—C12	1.223 (3)	C11—H11A	0.9700
O2—C15	1.220 (3)	C11—H11B	0.9700
N1—C15	1.385 (3)	C12—C13	1.430 (4)
N1—C12	1.400 (3)	C13—C14	1.331 (3)
N1—C11	1.475 (3)	C13—H13	0.9300
N2—C14	1.362 (3)	C14—H14	0.9300
N2—C15	1.376 (4)	C16—C17	1.509 (3)
N2—C16	1.465 (3)	C16—H16A	0.9700
C1—C6	1.369 (4)	C16—H16B	0.9700
C1—C2	1.382 (4)	C17—C18	1.365 (4)
C1—C11	1.513 (4)	C17—C22	1.375 (4)
C2—C3	1.370 (4)	C18—C19	1.384 (4)
C2—H2	0.9300	C18—H18	0.9300
C3—C4	1.391 (4)	C19—C20	1.381 (4)
C3—H3	0.9300	C19—H19	0.9300
C4—C5	1.371 (4)	C20—C21	1.380 (4)
C4—C7	1.527 (4)	C20—C23	1.538 (4)
C5—C6	1.383 (4)	C21—C22	1.382 (4)
C5—H5	0.9300	C21—H21	0.9300
C6—H6	0.9300	C22—H22	0.9300
C7—C8	1.465 (6)	C23—C25	1.472 (7)
C7—C9'	1.472 (10)	C23—C26	1.510 (7)
C7—C8'	1.483 (9)	C23—C26'	1.531 (11)
C7—C10	1.482 (7)	C23—C24	1.541 (6)
C7—C9	1.585 (6)	C23—C24'	1.544 (10)
C7—C10'	1.614 (9)	C23—C25'	1.545 (10)
C8—H8A	0.9600	C24—H24A	0.9600
C8—H8B	0.9600	C24—H24B	0.9600
C8—H8C	0.9600	C24—H24C	0.9600
C9—H9A	0.9600	C25—H25A	0.9600
C9—H9B	0.9600	C25—H25B	0.9600
C9—H9C	0.9600	C25—H25C	0.9600
C10—H10A	0.9600	C26—H26A	0.9600
C10—H10B	0.9600	C26—H26B	0.9600
C10—H10C	0.9600	C26—H26C	0.9600
C8'—H8'A	0.9600	C24'—H24D	0.9600
C8'—H8'B	0.9600	C24'—H24E	0.9600
C8'—H8'C	0.9600	C24'—H24F	0.9600
C9'—H9'A	0.9600	C25'—H25D	0.9600
C9'—H9'B	0.9600	C25'—H25E	0.9600
C9'—H9'C	0.9600	C25'—H25F	0.9600
C10'—H10D	0.9600	C26'—H26D	0.9600
C10'—H10E	0.9600	C26'—H26E	0.9600
C10'—H10F	0.9600	C26'—H26F	0.9600
			
C15—N1—C12	125.0 (2)	C12—C13—H13	119.8
C15—N1—C11	117.6 (2)	C13—C14—N2	122.6 (3)
C12—N1—C11	117.4 (2)	C13—C14—H14	118.7
C14—N2—C15	121.4 (2)	N2—C14—H14	118.7
C14—N2—C16	119.0 (2)	O2—C15—N2	122.1 (3)
C15—N2—C16	119.5 (2)	O2—C15—N1	122.0 (3)
C6—C1—C2	117.6 (3)	N2—C15—N1	115.9 (2)
C6—C1—C11	121.5 (3)	N2—C16—C17	113.8 (2)
C2—C1—C11	120.9 (3)	N2—C16—H16A	108.8
C3—C2—C1	120.8 (3)	C17—C16—H16A	108.8
C3—C2—H2	119.6	N2—C16—H16B	108.8
C1—C2—H2	119.6	C17—C16—H16B	108.8
C2—C3—C4	122.4 (3)	H16A—C16—H16B	107.7
C2—C3—H3	118.8	C18—C17—C22	117.2 (2)
C4—C3—H3	118.8	C18—C17—C16	120.0 (2)
C5—C4—C3	115.8 (3)	C22—C17—C16	122.7 (2)
C5—C4—C7	121.9 (3)	C17—C18—C19	121.9 (2)
C3—C4—C7	122.2 (3)	C17—C18—H18	119.0
C4—C5—C6	122.3 (3)	C19—C18—H18	119.0
C4—C5—H5	118.8	C20—C19—C18	121.5 (2)
C6—C5—H5	118.8	C20—C19—H19	119.3
C1—C6—C5	121.1 (3)	C18—C19—H19	119.3
C1—C6—H6	119.4	C21—C20—C19	116.1 (2)
C5—C6—H6	119.4	C21—C20—C23	121.7 (2)
C8—C7—C9'	129.8 (11)	C19—C20—C23	122.2 (2)
C8—C7—C8'	53.3 (7)	C20—C21—C22	122.3 (3)
C9'—C7—C8'	122.8 (12)	C20—C21—H21	118.8
C8—C7—C10	116.1 (6)	C22—C21—H21	118.8
C9'—C7—C10	31.3 (9)	C17—C22—C21	120.9 (2)
C8'—C7—C10	143.6 (7)	C17—C22—H22	119.5
C8—C7—C4	111.4 (3)	C21—C22—H22	119.5
C9'—C7—C4	115.3 (11)	C25—C23—C26	113.3 (6)
C8'—C7—C4	108.4 (6)	C25—C23—C26'	131.3 (5)
C10—C7—C4	107.6 (4)	C26—C23—C26'	56.2 (6)
C8—C7—C9	106.0 (5)	C25—C23—C20	111.5 (3)
C9'—C7—C9	73.3 (9)	C26—C23—C20	106.9 (3)
C8'—C7—C9	57.2 (8)	C26'—C23—C20	117.0 (5)
C10—C7—C9	104.2 (5)	C25—C23—C24	110.3 (5)
C4—C7—C9	111.2 (3)	C26—C23—C24	107.8 (5)
C8—C7—C10'	46.7 (7)	C26'—C23—C24	51.6 (5)
C9'—C7—C10'	95.9 (12)	C20—C23—C24	106.8 (3)
C8'—C7—C10'	97.6 (10)	C25—C23—C24'	49.9 (6)
C10—C7—C10'	71.6 (8)	C26—C23—C24'	141.5 (5)
C4—C7—C10'	114.8 (5)	C26'—C23—C24'	105.5 (8)
C9—C7—C10'	132.8 (6)	C20—C23—C24'	111.6 (5)
C7—C8—H8A	109.5	C24—C23—C24'	62.7 (7)
C7—C8—H8B	109.5	C25—C23—C25'	60.0 (6)
H8A—C8—H8B	109.5	C26—C23—C25'	56.1 (6)
C7—C8—H8C	109.5	C26'—C23—C25'	104.5 (7)
H8A—C8—H8C	109.5	C20—C23—C25'	110.7 (4)
H8B—C8—H8C	109.5	C24—C23—C25'	142.0 (5)
C7—C9—H9A	109.5	C24'—C23—C25'	106.7 (8)
C7—C9—H9B	109.5	C23—C24—H24A	109.5
C7—C9—H9C	109.5	C23—C24—H24B	109.5
C7—C10—H10A	109.5	H24A—C24—H24B	109.5
C7—C10—H10B	109.5	C23—C24—H24C	109.5
H10A—C10—H10B	109.5	H24A—C24—H24C	109.5
C7—C10—H10C	109.5	H24B—C24—H24C	109.5
H10A—C10—H10C	109.5	C23—C25—H25A	109.5
H10B—C10—H10C	109.5	C23—C25—H25B	109.5
C7—C8'—H8'A	109.5	H25A—C25—H25B	109.5
C7—C8'—H8'B	109.5	C23—C25—H25C	109.5
H8'A—C8'—H8'B	109.5	H25A—C25—H25C	109.5
C7—C8'—H8'C	109.5	H25B—C25—H25C	109.5
H8'A—C8'—H8'C	109.5	C23—C26—H26A	109.5
H8'B—C8'—H8'C	109.5	C23—C26—H26B	109.5
C7—C9'—H9'A	109.5	H26A—C26—H26B	109.5
C7—C9'—H9'B	109.5	C23—C26—H26C	109.5
H9'A—C9'—H9'B	109.5	H26A—C26—H26C	109.5
C7—C9'—H9'C	109.5	H26B—C26—H26C	109.5
H9'A—C9'—H9'C	109.5	C23—C24'—H24D	109.5
H9'B—C9'—H9'C	109.5	C23—C24'—H24E	109.5
C7—C10'—H10D	109.5	H24D—C24'—H24E	109.5
C7—C10'—H10E	109.5	C23—C24'—H24F	109.5
H10D—C10'—H10E	109.5	H24D—C24'—H24F	109.5
C7—C10'—H10F	109.5	H24E—C24'—H24F	109.5
H10D—C10'—H10F	109.5	C23—C25'—H25D	109.5
H10E—C10'—H10F	109.5	C23—C25'—H25E	109.5
N1—C11—C1	112.6 (2)	H25D—C25'—H25E	109.5
N1—C11—H11A	109.1	C23—C25'—H25F	109.5
C1—C11—H11A	109.1	H25D—C25'—H25F	109.5
N1—C11—H11B	109.1	H25E—C25'—H25F	109.5
C1—C11—H11B	109.1	C23—C26'—H26D	109.5
H11A—C11—H11B	107.8	C23—C26'—H26E	109.5
O1—C12—N1	120.1 (3)	H26D—C26'—H26E	109.5
O1—C12—C13	125.1 (3)	C23—C26'—H26F	109.5
N1—C12—C13	114.7 (2)	H26D—C26'—H26F	109.5
C14—C13—C12	120.4 (3)	H26E—C26'—H26F	109.5
C14—C13—H13	119.8		
			
C6—C1—C2—C3	0.1 (4)	C14—N2—C15—O2	179.4 (2)
C11—C1—C2—C3	179.8 (2)	C16—N2—C15—O2	−2.4 (4)
C1—C2—C3—C4	−0.5 (4)	C14—N2—C15—N1	−1.4 (3)
C2—C3—C4—C5	0.5 (4)	C16—N2—C15—N1	176.8 (2)
C2—C3—C4—C7	−178.1 (3)	C12—N1—C15—O2	178.7 (2)
C3—C4—C5—C6	−0.1 (4)	C11—N1—C15—O2	0.7 (4)
C7—C4—C5—C6	178.5 (3)	C12—N1—C15—N2	−0.5 (3)
C2—C1—C6—C5	0.3 (4)	C11—N1—C15—N2	−178.4 (2)
C11—C1—C6—C5	−179.4 (3)	C14—N2—C16—C17	−74.7 (3)
C4—C5—C6—C1	−0.3 (5)	C15—N2—C16—C17	107.0 (3)
C5—C4—C7—C8	124.6 (6)	N2—C16—C17—C18	147.9 (3)
C3—C4—C7—C8	−56.9 (6)	N2—C16—C17—C22	−34.4 (4)
C5—C4—C7—C9'	−74.5 (11)	C22—C17—C18—C19	0.1 (4)
C3—C4—C7—C9'	104.1 (11)	C16—C17—C18—C19	177.9 (2)
C5—C4—C7—C8'	67.6 (9)	C17—C18—C19—C20	−0.2 (4)
C3—C4—C7—C8'	−113.9 (9)	C18—C19—C20—C21	0.0 (4)
C5—C4—C7—C10	−107.1 (6)	C18—C19—C20—C23	179.7 (2)
C3—C4—C7—C10	71.4 (6)	C19—C20—C21—C22	0.3 (4)
C5—C4—C7—C9	6.5 (4)	C23—C20—C21—C22	−179.4 (3)
C3—C4—C7—C9	−175.0 (4)	C18—C17—C22—C21	0.1 (4)
C5—C4—C7—C10'	175.4 (10)	C16—C17—C22—C21	−177.6 (3)
C3—C4—C7—C10'	−6.0 (10)	C20—C21—C22—C17	−0.3 (4)
C15—N1—C11—C1	94.1 (3)	C21—C20—C23—C25	174.6 (6)
C12—N1—C11—C1	−84.1 (3)	C19—C20—C23—C25	−5.0 (7)
C6—C1—C11—N1	105.3 (3)	C21—C20—C23—C26	−61.1 (5)
C2—C1—C11—N1	−74.4 (3)	C19—C20—C23—C26	119.2 (5)
C15—N1—C12—O1	−177.5 (2)	C21—C20—C23—C26'	−0.9 (7)
C11—N1—C12—O1	0.4 (3)	C19—C20—C23—C26'	179.4 (7)
C15—N1—C12—C13	2.0 (3)	C21—C20—C23—C24	54.0 (5)
C11—N1—C12—C13	180.0 (2)	C19—C20—C23—C24	−125.6 (4)
O1—C12—C13—C14	177.8 (3)	C21—C20—C23—C24'	120.7 (8)
N1—C12—C13—C14	−1.7 (3)	C19—C20—C23—C24'	−59.0 (8)
C12—C13—C14—N2	0.0 (4)	C21—C20—C23—C25'	−120.5 (8)
C15—N2—C14—C13	1.7 (4)	C19—C20—C23—C25'	59.8 (8)
C16—N2—C14—C13	−176.6 (2)		
